# “NeuroVanguard”: a contemporary strategy in neuromonitoring for severe adult brain injury patients

**DOI:** 10.1186/s13054-024-04893-4

**Published:** 2024-04-01

**Authors:** Edith Elianna Rodriguez, Mario Zaccarelli, Elda Diletta Sterchele, Fabio Silvio Taccone

**Affiliations:** 1 Department of Intensive Care, Hopital Universitaire de Bruxelles (HUB), Université Libre de Bruxelles (ULB), Route de Lennik, 808, 1070 Brussels, Belgium; 2 School of Medicine and Health Sciences, Universidad del Rosario, Bogotá, Colombia; 3 Department of Surgical Sciences and Integrated Diagnostics, University of Genoa, Genoa, Italy; 4 Terapia Intensiva e del Dolore, Scuola di Anestesia Rianimazione, Università degli Studi di Milano, Milan, Italy

**Keywords:** Acute brain injury, Neuromonitoring, Integrated physiology, Individualized care

## Abstract

Severe acute brain injuries, stemming from trauma, ischemia or hemorrhage, remain a significant global healthcare concern due to their association with high morbidity and mortality rates. Accurate assessment of secondary brain injuries severity is pivotal for tailor adequate therapies in such patients. Together with neurological examination and brain imaging, monitoring of systemic secondary brain injuries is relatively straightforward and should be implemented in all patients, according to local resources. Cerebral secondary injuries involve factors like brain compliance loss, tissue hypoxia, seizures, metabolic disturbances and neuroinflammation. In this viewpoint, we have considered the combination of specific noninvasive and invasive monitoring tools to better understand the mechanisms behind the occurrence of these events and enhance treatment customization, such as intracranial pressure monitoring, brain oxygenation assessment and metabolic monitoring. These tools enable precise intervention, contributing to improved care quality for severe brain injury patients. The future entails more sophisticated technologies, necessitating knowledge, interdisciplinary collaboration and resource allocation, with a focus on patient-centered care and rigorous validation through clinical trials.

## The initial approach to the brain-injured patient

**Fig. 1:**
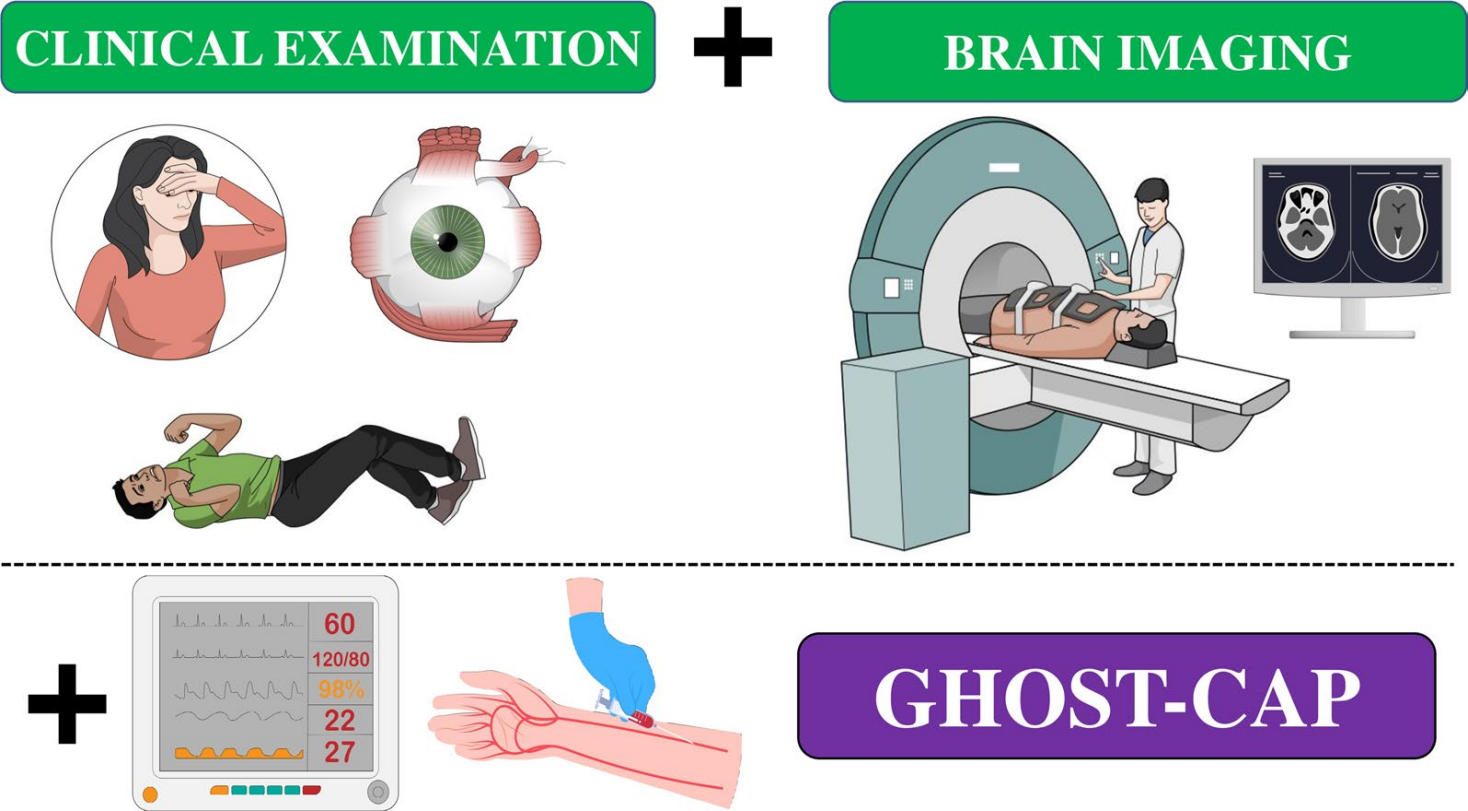
Initial assessment of the brain-injured patients, including clinical examination and brain imaging. For all patients, a rapid assessment of the presence of secondary systemic brain injuries using the GHOST-CAP acronym (i.e., via patient’ monitor and arterial gas analyses) is required

Severe acute brain injuries, whether resulting from trauma, ischemia or hemorrhage, remain a significant public health concern due to their association with high morbidity and mortality rates in healthcare systems worldwide [1, 2]. The severity of the initial primary injury is a crucial determinant of patient outcomes in this context [3, 4]. To gauge the extent of primary injury, clinicians rely on a combination of neurological examination and brain imaging (Fig. 1). The neurological examination serves as the cornerstone for assessing the consequences of acute brain injury on cerebral function [5], allowing to evaluate consciousness, verbal expression, motor function, sensory deficits and brainstem integrity. Furthermore, healthcare professionals frequently condense the initial neurological assessment into a set of selected clinical signs; these signs play a pivotal role in promptly quantifying the extent of neurological impairment, are easily replicable during subsequent evaluations and hold significant prognostic value. The most commonly utilized assessment tool is the Glasgow Coma Scale (GCS), including 3 items, which has been extensively validated in patients with traumatic brain injuries (TBI) [6]. Furthermore, a modified GCS has been adapted to formulate the World Federation of Neurological Societies (WFNS) scale, specifically employed for patients presenting with non-traumatic subarachnoid hemorrhage (SAH) on admission [7]. In patients with ischemic or hemorrhagic stroke, the National Institutes of Health Stroke Scale (NIHSS) is particularly valuable as it uses 11 items, each designed to assess specific aspects of neurological function, such as consciousness, eye movement, visual fields, facial palsy, motor function, limb ataxia, sensory loss, language abnormalities and inattention, with higher scores indicating more severe deficits [8].

In conjunction with clinical evaluation, brain imaging plays a pivotal role in quantifying the extent of brain damage and its correlation with clinical signs. Computed tomography (CT) scans provide rapid assessment of intracranial pathology, such as the presence of hemorrhage or ischemic injuries, skull fractures, acute hydrocephalus or brain edema [9]. The recent evolution of CT scanning, specifically the integration of CT angiography (CTA) and CT perfusion (CTP) using intravenous contrast agents and specific acquisition techniques, allows for high-resolution imaging of the cerebral arteries and veins, and provides detailed information about vascular anatomy and the presence of abnormalities (i.e., arterial stenosis, aneurysms or vascular malformations) [10, 11]. Also, CTA and CTP offer critical insights into tissue viability, identifying areas of hypoperfusion and distinguishing salvageable brain tissue from irreversibly damaged regions [10, 11]. Conversely, magnetic resonance imaging (MRI) provides superior anatomical resolution, rendering it particularly advantageous in cases involving ischemic damage or diffuse axonal injuries [12]; however, as MRI typically requires a longer acquisition time, it is usually selected as an alternative to CT scan.

## Which are the goals of treatment and how monitoring can help?

The overarching goal of managing such critical conditions is to prevent or mitigate secondary brain injuries. Secondary brain injuries are those that occur as a consequence of the initial insult (e.g., seconds, minutes or hours after the initial injury has occurred) and result from a cascade of complex pathological processes, encompassing a range of systemic and cerebral events that can exacerbate the primary injury and lead to poorer outcomes [13]. In this manuscript, when addressing secondary brain injuries, we defined “systemic” abnormalities as those originating outside the cranial cavity that can lead to brain dysfunction, while “cerebral” abnormalities those dependent on a primary intracranial mechanism. However, we acknowledge that this classification may be somewhat artificial in clinical practice. For instance, hypoglycemia, typically considered a systemic secondary brain injury, may increase the occurrence of seizures, which are typically considered as a secondary cerebral brain injury.

While the assessment of brain injury severity, whether on admission or during the hospital stay, heavily relies on clinical examination, it is essential to acknowledge the inherent limitations of this approach to consistently detect secondary injuries. Firstly, in patients admitted with altered levels of consciousness, clinical examination may yield limited or unreliable information, particularly in cases of drug or alcohol intoxication, where sedatives or muscle paralyzers are administered [14]. Secondly, the identification of subtle neurological changes can pose a significant challenge, especially in individuals with preexisting neurological deficits, psychiatric/behavioral disorders or comorbid conditions. Thirdly, the manifestation of new neurological impairments is non-specific to the underlying mechanism, thereby restricting the immediate initiation of appropriate therapies in all cases. Moreover, the clinical examination can only assess the current functionality of the neurological systems, while it cannot predict which functions may be regained over time. Lastly, clinical examination can prove to be demanding for non-neurologists, potentially leading to an underestimation of ongoing brain damage [15].

Consequently, there is a growing recognition within the medical community of the imperative need for supplementary tools to enhance the early and accurate detection of secondary brain injuries in such patients. The role of invasive and noninvasive neuromonitoring in brain-injured patients is crucial for assessing neurological status, guiding treatment decisions and predicting outcomes. However, the utilization of these techniques remains limited due to the lack of robust evidence supporting their efficacy in improving outcomes. Furthermore, they are often employed in isolation rather than in combination, which may restrict their ability to comprehensively assess the complexity of brain dysfunction in this context. Addressing the considerable variability in the utilization and implementation of such monitoring tools, we present the “NeuroVanguard” concept in this viewpoint as a contemporary strategy in neuromonitoring that warrants evaluation and consideration for severe adult brain-injured patients. Importantly, the majority of the existing literature supporting certain proposed statements is derived from studies conducted in TBI patients, with limited data available for other types of brain injury. Therefore, while we have discussed the NeuroVanguard concept as relevant to all forms of acute brain injury, additional research is necessary to thoroughly investigate most of our hypotheses and proposals beyond the scope of TBI.

## The first domain of neuromonitoring: tackling systemic secondary brain injuries

The identification of systemic secondary brain injuries is relatively straightforward within the intensive care unit (ICU) setting. Specifically, patient monitoring and the results obtained from arterial gas analyses provide sufficient data to recognize variables that can significantly impact brain function in this context (Fig. 1). To assist healthcare providers in this critical task, a mnemonic acronym, “GHOST-CAP,” has been introduced [13]. This acronym encompasses glucose, hemoglobin, oxygen, sodium, temperature, comfort, arterial blood pressure and carbon dioxide levels. While these variables play pivotal roles in maintaining cerebral homeostasis, there remains ongoing debate regarding the ideal ranges for their management in this context (Table 1). Nonetheless, understanding these values is essential for detecting any deterioration in the neurological status of brain-injured patients [16, 17] and should be widely implemented, according to local resources.

**Table 1:** Different components of the GHOST-CAP acronym

		Targets	Indivualized targets
G	Glucose	80–180 mg/dL	CMD
H	Hemoglobin	> 7 g/dL	PbtO_2_
O	Oxygen	PaO_2_ = 80–120 mmHg	PbtO_2_
S	Sodium	135–145 mEq/L	ICP
T	Temperature	< 38.0 °C	ICP, PbtO_2_, CMD
C	Comfort	No pain and agitation	ICP, PbtO_2_, CMD
A	Arterial pressure	MAP > 80 mmHg	ICP, PbtO_2_, CMD
P	PaCO_2_	35–40 mmHg	ICP, PbtO_2_, CMD

*CMD* cerebral microdialysis, *PbtO*_*2*_ brain oxygen pressure, *ICP* intracranial pressure, *MAP* mean arterial pressure

For instance, consider a scenario where a traumatic brain injury (TBI) patient exhibits a progressive reduction in the GCS score, transitioning from 13 to 9; simultaneously, there is a drop in sodium levels from 139 to 128 mmol/L or the core temperature rises from 37.1 to 38.9 °C. In this situation, clinicians should carefully consider either to perform brain imaging or to correct these systemic disturbances and observe whether patient's initial clinical examination could be restored. This latter approach may obviate the need for immediate transport of the patient to the CT scan or MRI suite; if the clinical examination remains impaired after addressing these abnormalities, additional diagnostic procedures would be warranted.

**Fig. 2:**
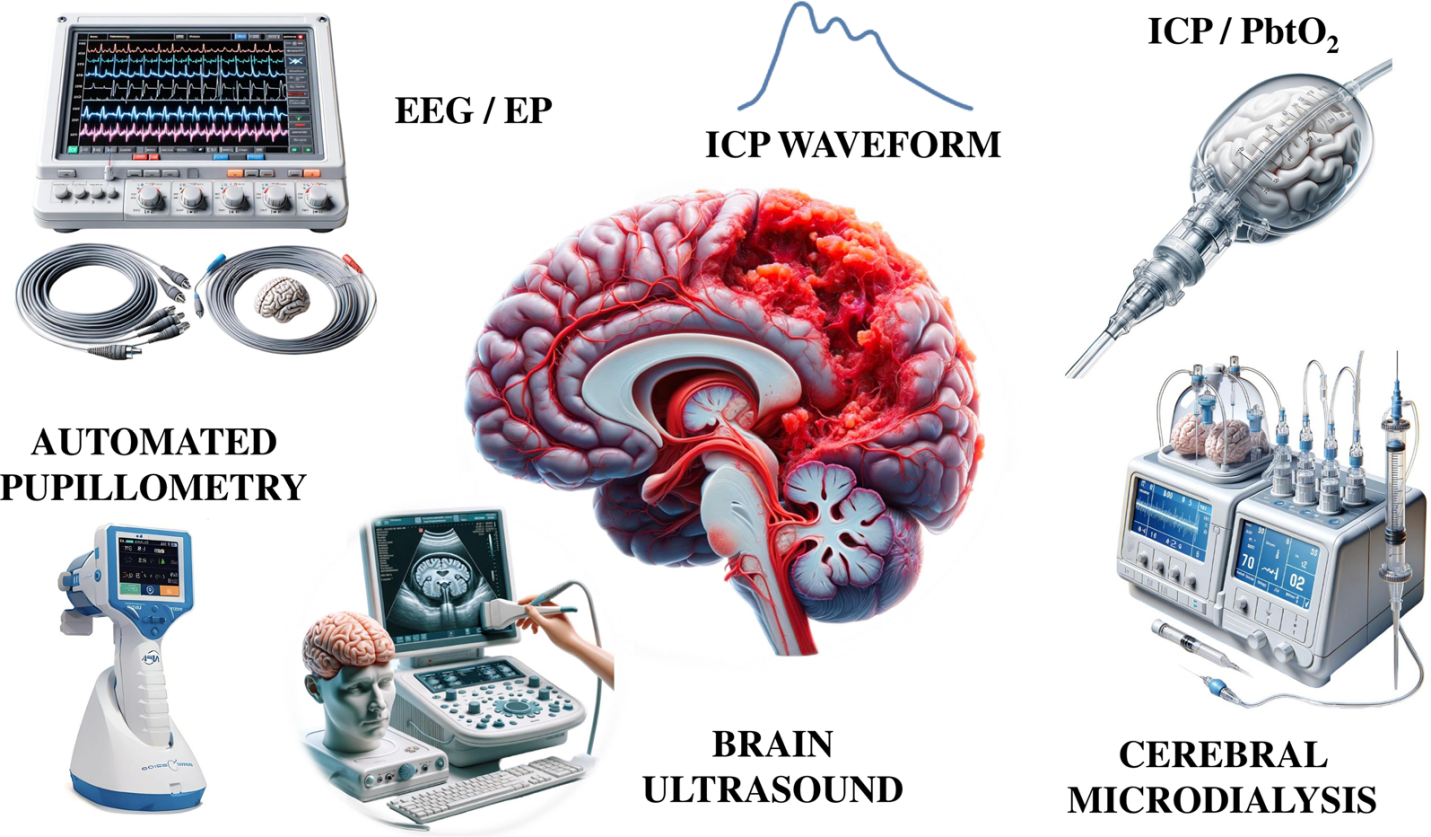
A comprehensive monitoring of brain function after an acute brain injury, including electroencephalography (EEG) and/or evoked potentials (EP), noninvasive assessment of intracranial pressure (ICP) waveform, invasive ICP and brain oxygen pressure (PbtO_2_) measurements, cerebral microdialysis, brain ultrasound (including cerebral blood flow velocities and optic nerve sheath diameter) and automated pupillometry

In the NeuroVanguard approach, to address the limitations associated with the use of fixed thresholds for initiating therapeutic interventions based on individual physiological variables, the utilization of specific monitoring tools becomes relevant in optimizing treatment decisions tailored to each patient's unique needs. Notably, intracranial pressure (ICP) monitoring, brain oxygenation and metabolic monitoring are critical in this regard [18]. For example, ICP monitoring serves as a valuable guide in determining the need for interventions such as sedation management, sodium level adjustments, temperature control, MAP optimization and regulation of pH via PaCO_2_ manipulation [19]. Similarly, brain oxygenation monitoring plays a crucial role in individualizing target ranges for parameters including oxygen levels, MAP, pH, hemoglobin concentration and temperature [20–22] (Table 1). Despite the significance of monitoring systemic variables, the integration of multimodal neuromonitoring, which may involve invasive and noninvasive techniques (Fig. 2), offers a comprehensive approach to enhancing the customization of therapeutic targets [18]. This approach not only facilitates precise treatment but also contributes to the overall enhancement of care quality for patients with severe brain injuries.

## The second domain: understanding the limitations of intracranial pressure monitoring

Intracranial pressure (ICP) monitoring plays a pivotal role in the management of patients with TBI and SAH, while its role in patients with hemorrhagic stroke remains more debated [23]. Elevated ICP serves as a well-established predictor of unfavorable outcomes in such patients, underscoring the critical importance of early detection and intervention [24]. However, it is crucial to emphasize that elevated ICP is not a diagnosis in itself but rather an “alert” signal, which does not provide specific information regarding the underlying mechanisms responsible for the increased pressure within the cranial vault. Elevated ICP can manifest due to cerebral edema, hydrocephalus (i.e., abnormal accumulation of cerebrospinal fluid, CSF, within the brain), hyperemia (i.e., excessive arterial blood brain volume, frequently associated with conditions like hypercarbia or hypertension), altered cerebral venous return (i.e., venous sinus thrombosis or jugular compression) or mass effects (i.e., space-occupying, such as hematomas, tumors or abscesses) [25]. These lesions can exert pressure on surrounding brain tissue and exhaust brain compensatory mechanisms, such as the rostral displacement of CSF and the reduction in the venous blood volume, ultimately leading to decreased intracranial compliance [26].

Although ICP monitoring has a critical role in severe adult brain-injured patients, its impact on patients’ outcome remains controversial. In one study, ICP monitoring was linked to unfavorable neurological outcomes at the 6-month, along with complications such as respiratory issues, infections, prolonged ICU length of stay and extended mechanical ventilation duration [27]. In a separate prospective observational study, substantial variation in the utilization of ICP monitoring was observed across different medical centers [28]; notably, patients subjected to ICP monitoring displayed lower 6-month mortality rates compared to those who were not monitored. The reluctance to universally embrace ICP monitoring may, in part, be attributed to the outcomes of a randomized trial, published in 2012, which failed to demonstrate the superiority of ICP-guided treatment over neurological assessment and serial CT-based treatment approaches in terms of achieving favorable neurological recovery [29]. It is worth noting that the trial employed a therapeutic algorithm for managing ICP different than recent recommendations [30], which only one-third of patients experienced intracranial hypertension, and was conducted in South America, thereby limiting the generalizability of its findings.

Additional limitations of ICP monitoring merit consideration in the clinical management of acute brain injuries. First and foremost, protocolized therapy is based on specific ICP threshold; the widely adopted threshold of ICP greater than 22 mmHg serves as a trigger for therapeutic interventions aimed at reducing ICP after TBI [31]. However, this approach lacks insight into individual variations in brain tolerance to ICP values and has not been validated in other forms of brain injury. Patients may exhibit differing tolerances, with some able to withstand higher ICP levels without adverse consequences, while others may experience detrimental effects at lower ICP levels (i.e., between 15 and 25 mmHg). These variations in tolerance are influenced by factors such as cerebral perfusion pressure and cerebral reserve, underscoring the inadequacy of rigid adherence to a single ICP threshold [32]. Secondly, the duration of exposure to elevated ICP, often referred to as ICP “dose,” is a critical consideration. Prolonged periods of ICP within the range of 15–25 mmHg can lead to more substantial cerebral damage than transient elevations [33]; as such, fixed ICP thresholds fail to account for this essential temporal factor. Thirdly, different patient populations, including adults, children and the elderly, may manifest distinct responses to elevated ICP [33]. Therefore, a uniform approach based on a single threshold may not be suitable for diverse groups of patients. Finally, it is imperative to recognize that brain injury is a complex process influenced by a multitude of factors beyond ICP; solely focusing on ICP thresholds may lead to an oversimplified view of the broader context of brain injury [34].

In light of these limitations, healthcare providers must understand that elevated ICP serves as a significant clinical finding associated with adverse outcomes. However, it represents a non-specific marker of a potentially serious underlying issue. Achieving precise diagnosis and treatment necessitates a deeper comprehension of the specific cause or mechanism responsible for the elevated ICP, which can vary among individual patients. Given the intricate interplay of these factors, it becomes evident that monitoring ICP in isolation is insufficient to guide therapy effectively. In the NeuroVanguard approach, the integration of multimodal neuromonitoring becomes imperative to interpret ICP at bedside. This comprehensive approach accounts for the dynamic and multifaceted nature of acute brain injuries, enabling healthcare providers to make informed decisions and optimize patient care.

## The third domain: focusing on brain compliance

**Fig. 3:**
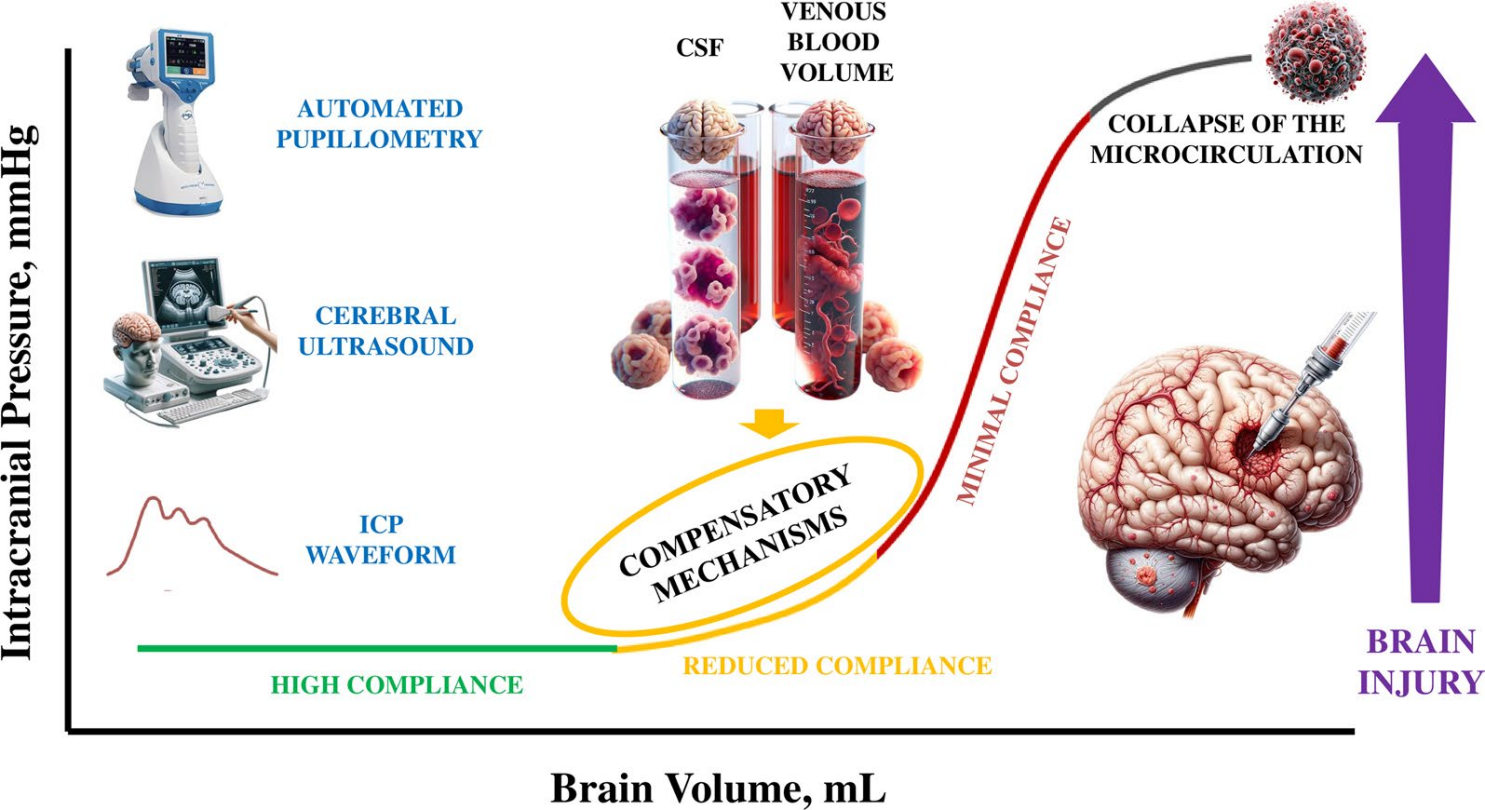
The correlation between intracranial pressure (ICP) and brain volume serves as a valuable metric for assessing brain compliance. In instances characterized by high compliance (green line), alterations in brain volume do not precipitate a corresponding elevation in ICP. However, as brain volume continues to expand, compensatory mechanisms come into play; these mechanisms encompass the displacement of cerebrospinal fluid (CSF, i.e., rostral movement outside the cranial vault) and the reduction in the venous blood volume. Over time, these compensatory processes become progressively depleted, leading to a transition from a state of reduced compliance (orange line) to minimal compliance (red line). In this latter state, even minor fluctuations in brain volume can elicit significant increases in ICP. The integration of automated pupillometry, cerebral ultrasound and evaluation of ICP waveform holds the potential to aid in gauging the extent of brain compliance, thereby facilitating a more comprehensive understanding of cerebral dynamics

Together with the implementation of ICP and cerebral perfusion pressure (CPP) monitoring in severe brain-injured patients, healthcare providers should also assess the relationship between ICP and intracerebral volume (Fig. 3). Drawing from previous studies that have identified a “safe” range for ICP below 15 mmHg and a “danger” zone for ICP exceeding 25 mmHg [33], the modern utilization of ICP should involve a deeper exploration of ICP values falling within the 15–25 mmHg range, with a focus on their association with brain compliance. Nonetheless, in the case of patients who have undergone decompressive craniectomy, it is advisable to contemplate this approach even for ICP values below 15 mmHg [35].

In cases where ICP values correlate with high compliance, it is expected that brain hemodynamics will remain within safe parameters, and brain perfusion will remain unaltered. However, when brain compliance begins to deteriorate, triggering compensatory mechanisms, noninvasive neuromonitoring can provide valuable insights into changes in brain compliance. As elegantly proposed by Godoy et al. [26], noninvasive neuromonitoring tools could be valuable in this context. While often regarded as predictors of invasive ICP, the findings obtained through automated pupillometry, cerebral ultrasound and ICP waveform analysis should be viewed as complementary to ICP values and should be integrated to enhance our comprehension of ICP [36]. As such, reduction in the Neurological Pupil Index (NPi) to less than 3, as measured through automated pupillometry, may indicate impairment of the mesencephalon due to cerebral edema, affecting brainstem function [37]. Additionally, reductions in the diastolic velocity of the middle cerebral artery below 20 cm/s or an increase in the pulsatility index above 1.4, as determined through cerebral ultrasound, can serve as surrogates for compromised cerebral hemodynamics resulting from reduced brain compliance [38]. Cerebral ultrasound can also be employed to assess the optic nerve sheath diameter (ONSD); if ONSD measurements exceed 6.0 mm, it may suggest that reduced cerebral compliance is impacting CSF circulation, leading to fluid accumulation around the optic nerve and subsequent ONSD enlargement [39]. Furthermore, noninvasive graphic assessment of the ICP waveform, either performed visually or using a dedicated technology, is a valuable tool [40]; an increase in the P2 waveform (representing the tidal wave, reflective of the arterial pulse within the brain parenchyma) relative to P1 (representing the percussion wave, attributable to arterial pulsations) suggests reduced cerebral compliance and the exhaustion of compensatory mechanisms.

## The fourth domain: focus on brain oxygenation and metabolism

In the complex and dynamic landscape of managing severely brain-injured patients, one alternative approach to brain compliance assessment is the monitoring of brain oxygenation, such as with PbtO_2_ catheters [41]; PbtO_2_ measures the partial pressure of oxygen within brain tissue and provides a dynamic evaluation of cerebral oxygenation, which is contingent upon cerebral blood flow and the arteriovenous oxygen difference (i.e., arterial oxygen content, oxygen consumption and oxygen diffusibility) [42]. One of the key advantages of PbtO_2_ monitoring is its capability to detect alterations in tissue oxygenation before they reach a point of irreversibility, as well as identifying instances of tissue hypoxia even in the absence of elevated ICP.

This proactive approach enables early intervention, preventing oxygen deprivation and potentially minimizing the risk of secondary brain injury. For instance, a decrease in PbtO_2_ levels may signal compromised oxygen supply to the brain, prompting healthcare providers to take immediate action to improve oxygenation [43]. In patients with initially low PbtO_2_ levels, optimizing CPP, administering a higher-oxygen therapy or providing red blood cell transfusion can lead to a substantial enhancement in tissue oxygenation [44]. Furthermore, PbtO_2_ monitoring allows for a more nuanced assessment of ICP impact on brain tissue. On the one side, low PbtO_2_ levels may indicate a potential mismatch between oxygen demand and supply in patients with borderline ICP values (i.e., 15–25 mmHg); this information can guide clinicians in tailoring interventions to optimize oxygenation by lowering ICP values [45]. On the other side, PbtO_2_ monitoring can also help tolerating high ICP levels in the context of patient awakening; brain-injured patients who have been sedated as part of their treatment may undergo periods of awakening during their recovery process. This transition can be challenging, as it may lead to increases in ICP due to various factors, including arousal-related metabolic demands; conventional practice often dictates strict adherence to low ICP thresholds (i.e., ICP < 20 mmHg) to prevent secondary cerebral brain injury. However, during the awakening phase, maintaining ICP within such narrow limits can be impractical and potentially counterproductive. This is where PbtO_2_ monitoring offers a more nuanced perspective; if PbtO_2_ levels remain within acceptable ranges despite a modest increase in ICP (i.e., ICP reaching 20–25 mmHg), it suggests that the brain is tolerating this elevation without significant oxygen deprivation [46]. In such cases, clinicians can cautiously allow for a temporary elevation in ICP during the awakening process, avoiding unnecessary interventions that might hinder progress.

However, the individualization of therapies based on the combination of ICP and PbtO_2_ also necessitates clinical validation and have several limitations (see *next paragraph*). A systematic review, primarily comprising 15 studies (involving a total of 37,245 patients), although predominantly observational with an overall low quality of evidence, indicated that the use of combined ICP/PbtO_2_-guided therapy, as opposed to ICP-guided therapy alone, was significantly associated with a higher likelihood of achieving a favorable neurological outcome and improved hospital survival [47]. In a phase II randomized trial conducted across 10 neuro-ICUs in the USA, a management protocol incorporating continuous PbtO_2_ monitoring alongside ICP monitoring demonstrated a notable reduction in the duration of brain tissue hypoxia among severe TBI patients, in comparison with those monitored for ICP alone [48], with a nonsignificant improvement in the proportion of patients achieving a favorable neurological outcome at 6 months. In a phase III randomized study involving 318 TBI patients, the addition of PbtO_2_ monitoring did not lead to a reduction in the proportion of patients experiencing poor neurological outcomes when compared to those monitored solely for ICP [49]. Furthermore, a higher incidence of intracerebral hematomas associated with catheter placement was observed in the ICP/PbtO_2_ group. Notably, there were variations in the protocols employed to optimize PbtO_2_ between these two studies, particularly concerning the timing of administering hyperoxia; these discrepancies in treatment approaches may partially account for some disparities in the reported outcomes. Additionally, differences in patient selection and specific therapeutic interventions chosen could contribute to the differing outcomes observed in the ICP monitoring group between these two studies, underlying the need for larger randomized trials in this context.

Moreover, as PbtO_2_ may also have some limitations, such as to ensure the appropriateness of catheter placement into a “high-risk” area (i.e., when the probe is inserted into healthy tissue, it would not yield pertinent information to guide therapeutic interventions), the potential reduced accuracy of measurements (i.e., after 7–10 days of monitoring) or the use of a specific cutoff (i.e., < 20 mmHg) to identify tissue hypoxia and initiate therapies, cerebral microdialysis has emerged as a valuable tool in achieving this individualization by providing real-time biochemical data from within the brain interstitial tissue [50]. Using this technique, it is possible to measure small molecules, including metabolites and markers of cellular distress, such as glucose, lactate, pyruvate, glutamate and glycerol. An elevation in the lactate-to-pyruvate ratio (LPR) in conjunction with reduced glucose and pyruvate levels can indicate a “hypoxic” state, offering valuable insights into the impact of ICP and/or PbtO_2_ levels on brain metabolic function [51]. For instance, if an increase in ICP to 18 mmHg coincides with low glucose, elevated LPR and reduced PbtO_2_ values, it may warrant specific interventions aimed at rectifying the imbalance between oxygen delivery and consumption. Conversely, certain patients may tolerate slightly elevated ICP or borderline PbtO_2_ values (i.e., 15–19 mmHg) without experiencing metabolic compromise. In such cases, healthcare providers can exercise discretion in avoiding aggressive interventions that may be unnecessary and carry the potential for unintended consequences. Importantly, there is currently a lack of well-designed prospective studies demonstrating the feasibility of tailoring therapies in brain-injured patients based on cerebral microdialysis data. Furthermore, certain metabolic abnormalities, such as those associated with mitochondrial dysfunction and/or hyperglycolysis, lack specific and validated interventions [52].

## The fifth domain: potential application and limitations

**Fig. 4:**
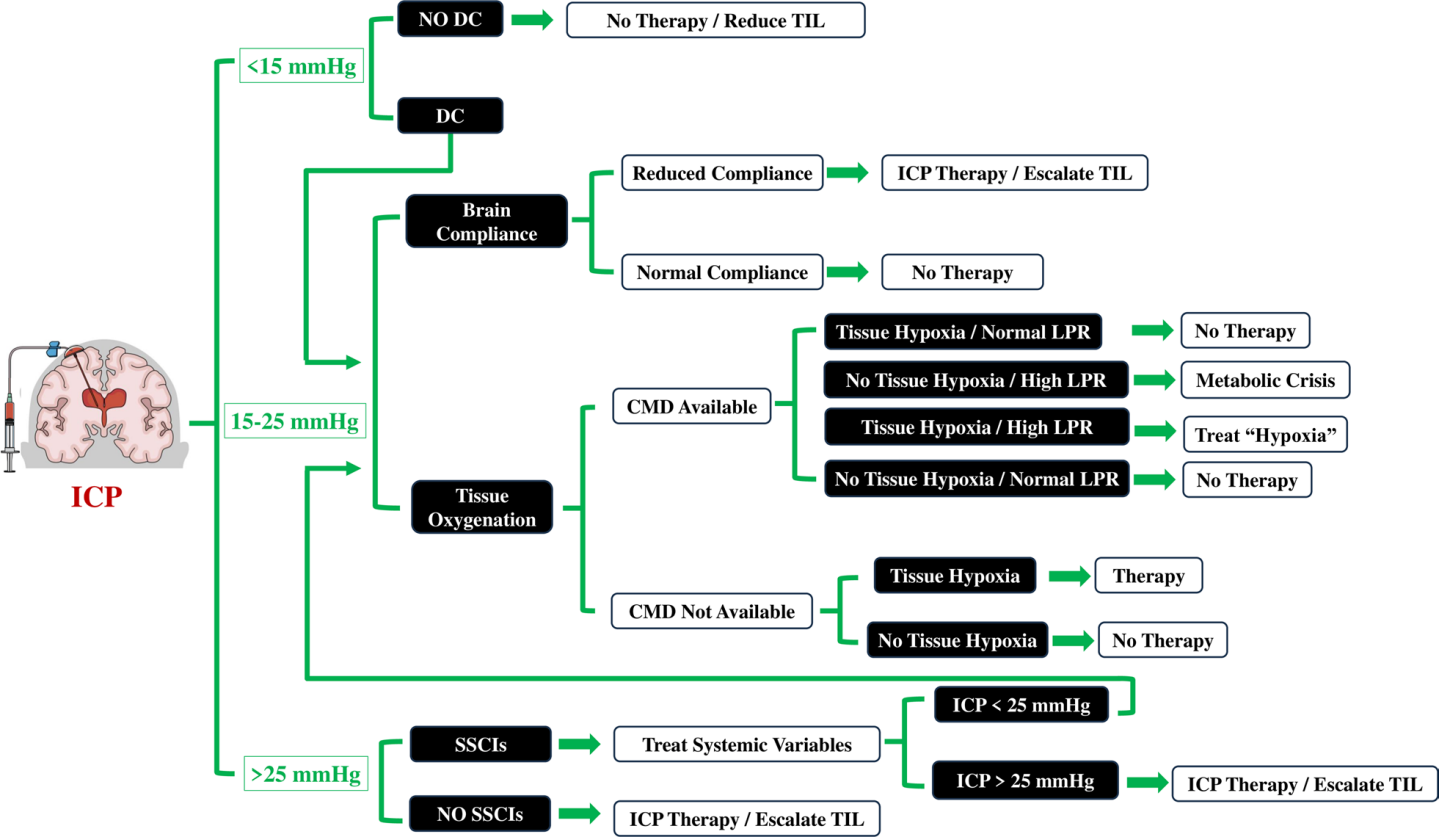
A diagnostic and therapeutic algorithm for severe brain-injured patients, initially guided by intracranial pressure (ICP) values, with subsequent individualized assessment of brain compliance or oxygenation for patients with ICP values ranging between 15 and 25 mmHg. At the end of each potential decision, the algorithm should circle back to ICP values. *DC* decompressive craniectomy, *TIL* therapy intensity level, *CMD* cerebral microdialysis, *LPR* lactate-to-pyruvate ratio, *SSBI* secondary systemic brain injuries

The field of neuromonitoring in brain-injured patients has witnessed remarkable advancements over the years, leading to a deeper understanding of the complex interplay between brain function and critical care. In the NeuroVanguard approach, by combining noninvasive tools, such as those assessing brain compliance, with invasive tools monitoring tissue hypoxia, in conjunction with ICP monitoring, clinicians can develop a comprehensive care protocol that enables the customization of therapeutic interventions within the “gray zone” of ICP values, typically ranging from 15 to 25 mmHg, as illustrated in Fig. 4.

The NeuroVanguard approach represents therefore a paradigm shift toward individualized ICP management strategies for brain-injured patients. Unlike the traditional "stepwise" approach to intracranial pressure (ICP) management, which relies on fixed thresholds to trigger interventions, NeuroVanguard would enable clinicians to assess the tolerance of brain hemodynamics or oxygenation to ICP values on an individual basis, facilitating the selection of optimal ICP thresholds to initiate therapy. By recognizing the heterogeneity in patient responses and the dynamic nature of brain physiology, NeuroVanguard aims therefore to promote personalized care pathways.

However, there are also several limitations to acknowledge. First, while this approach holds promise for enhancing the accuracy and precision in identifying potentially critical conditions involving altered cerebral hemodynamics and oxygenation, it is imperative to validate the feasibility and effectiveness of this strategy in clinical practice. This validation should include a comparison to the existing practices of using ICP alone or relying on clinical examination and repeated CT scans, as previously documented [53]. As such, the Neurovagard concept should not be considered as “standard of care” in the management of brain-injured patients. Moreover, there is a paucity of well-conducted studies demonstrating that therapies aimed at reducing ICP can be guided effectively by noninvasive neuromonitoring techniques. Second, clinical examination, even if limited, should always be incorporated into the assessment of severe brain-injured patients. In cases where clinical deterioration is observed, the NeuroVanguard approach remains invaluable for discerning the underlying mechanisms, such as secondary cerebral brain injuries, loss of brain compliance or tissue hypoxia. This approach aids in selecting appropriate additional diagnostic assessments (e.g., brain imaging or angiography) and tailoring therapies accordingly. Third, the majority of data supporting the NeuroVanguard concept stems from studies conducted in TBI patients, with limited evidence available for other forms of brain injuries. Therefore, further research is warranted to validate the effectiveness and utility of all monitoring tools included in the NeuroVanguard approach across various types of brain injuries. Fourth, we did not consider EEG findings and the estimation of optimal CPP in this approach. While conventional EEG remains an effective method for detecting non-convulsive seizures and assessing impending focal ischemia after SAH [54], its practical use for continuous bedside monitoring is currently limited by the requirement for expert interpretation from a certified neurophysiologist. However, quantitative EEG (qEEG, i.e., based on the use of mathematical algorithms to analyze raw EEG data, providing numerical metrics that can be readily interpreted by healthcare providers) offers promise as a more accessible and objective tool that can aid in assessing the depth of sedation [55]; further research and evaluation of qEEG in sedated brain-injured patients are warranted to determine its utility and potential impact on their global management. The identification of optimal CPP has been based on the lowest Pressure Reactivity Index (PRx), i.e., CPP ranges where cerebrovascular autoregulation is most effective, ensuring that the brain receives adequate blood flow while avoiding cerebral hypoperfusion or hyperperfusion [56]. While targeting optimal CPP using such approach has been studies in a phase II randomized trial including only TBI patients [57], CPP was within target in less than half of assessments, CPP was not available in 24% of readings, and clinicians did not apply recommended CPP targets in 11–33% of cases when optimal CPP was > 80 mmHg. Although the concept of optimal CPP based on the PRx index holds promise for improving neurocritical care, its implementation still faces several limitations and challenges. Additionally, other methods of monitoring brain oxygenation, such as those utilizing near-infrared spectroscopy (NIRS) technology or jugular bulb saturation (SjvO_2_), were not taken into account due to various limitations and reduced accuracy [58]. Finally, the adoption of advanced neuromonitoring techniques is often limited in countries with fewer resources due to various factors, such as high costs, lack of infrastructures, training programs and maintenance, making them inaccessible to healthcare facilities in these settings with constrained budgets.

**Fig. 5:**
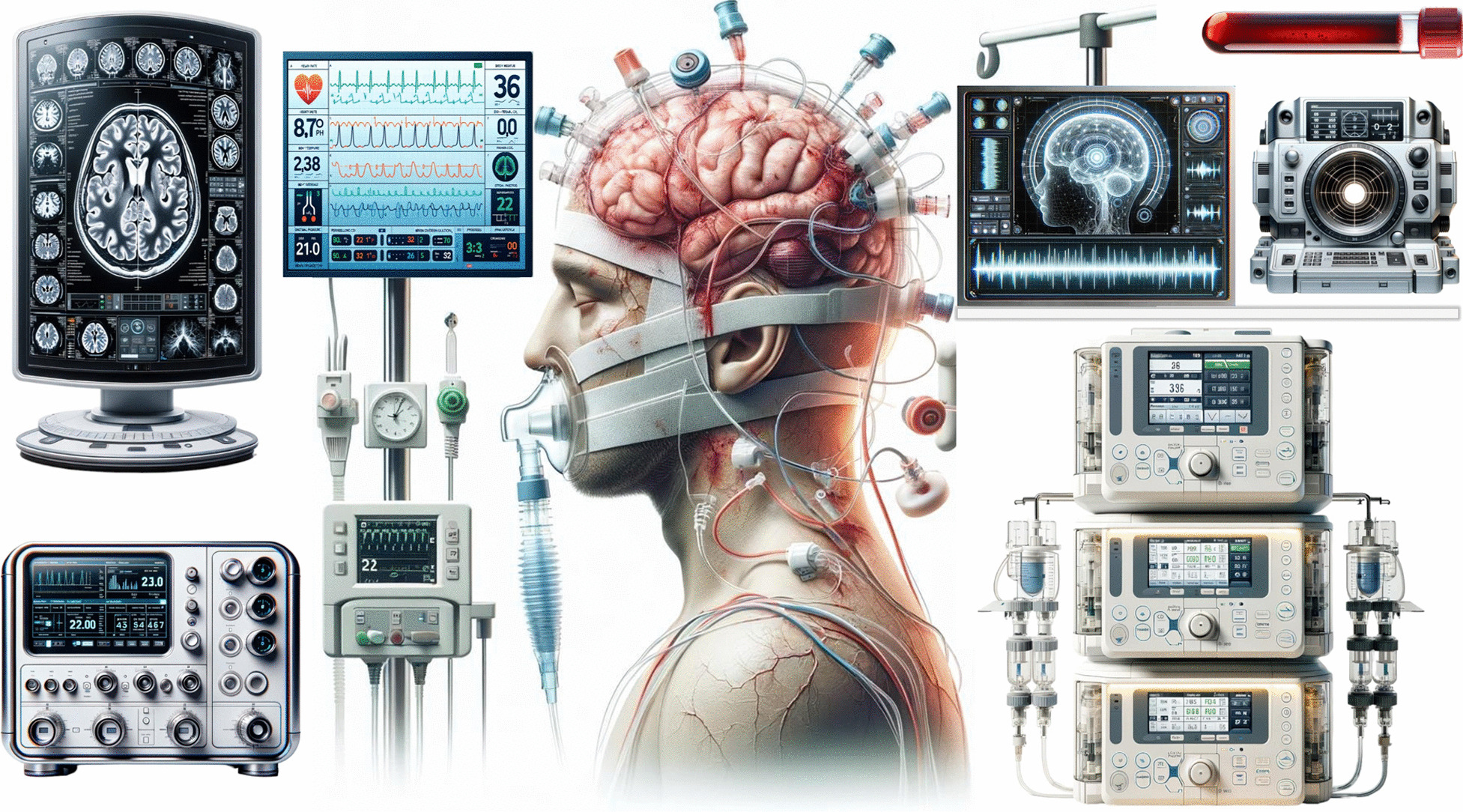
A futuristic and comprehensive neuromonitoring in brain-injured patients, encompassing (clockwise order from the top left) advanced bedside neuroimaging, systemic hemodynamics, quantitative electroencephalography, biomarkers, continuous pupillometry, personalized anesthetic management, intracranial pressure, brain oxygenation and metabolism

As technology continues to evolve, we can anticipate even more sophisticated tools and approaches that provide unprecedented accuracy and precision in monitoring and managing these patients (Fig. 5), resulting in future changes of the NeuroVanguard approach, accordingly. The field of neuroimaging is advancing rapidly, with innovations such as functional magnetic resonance imaging (fMRI), diffusion tensor imaging (DTI) and positron emission tomography (PET) offering insights into brain connectivity, white matter integrity and metabolic activity [59]. These imaging modalities will probably play a crucial role in assessing brain function and guiding therapeutic decisions. Future advancements may lead to noninvasive or minimally invasive techniques that provide real-time data on pupillary function, cerebral perfusion, oxygenation and metabolism. These technologies could reduce the need for invasive procedures and minimize the associated risks. Integration of Artificial Intelligence (AI) and machine learning algorithms will become integral to neuromonitoring. These tools will analyze vast datasets and assist clinicians in interpreting complex information, identifying patterns and making evidence-based decisions in real time, as it has already been suggested for EEG interpretation [60]. The development of reliable biomarkers and molecular profiling techniques will enable clinicians to detect subtle changes in brain physiology and predict patient outcomes more accurately. These biomarkers may extend beyond traditional biochemical markers and include genetic, proteomic and metabolomic data. Miniaturized sensors and wearable devices will allow for continuous monitoring of neurological parameters even after patients leave the ICU [61]. This extended monitoring will facilitate early detection of complications and better long-term management. The integration of telemedicine platforms will enable remote monitoring of brain-injured patients, providing expert guidance and support to healthcare providers in resource-limited settings.

While these technological advancements hold immense promise, they will also require a concerted effort in several key areas to fully realize their potential. As technology becomes more sophisticated, healthcare providers will need to acquire and maintain the knowledge and skills necessary to interpret and utilize the vast amount of data generated by neuromonitoring systems. Continuous education and training programs will be essential to ensure that clinicians are proficient in understanding and acting upon the information provided by these advanced tools [62]. Moreover, interdisciplinary collaboration will become increasingly important. Neurocritical care teams will need to work closely with neurologists, neurosurgeons, radiologists and data scientists to make sense of the multifaceted data generated by neuromonitoring. This collaborative approach will enhance decision-making and patient outcomes. The integration of cutting-edge technology into clinical practice comes with economic considerations. Hospitals and healthcare systems will consider to invest in state-of-the-art monitoring equipment, data infrastructure and skilled personnel to effectively implement these advancements. Budget allocation and resource management will play a crucial role in ensuring equitable access to these technologies. Additionally, healthcare policies and reimbursement structures may need to evolve to accommodate the cost of advanced neuromonitoring tools. Crucially, investments and reimbursement policies should be guided by high-quality data demonstrating the relevance of such monitoring tools in enhancing the quality of care and improving patient outcomes. Health economic studies will be required to assess the cost-effectiveness of these interventions and their impact on long-term outcomes. The wealth of data generated by various neuromonitoring modalities also poses a challenge in terms of integrating and interpreting this information comprehensively; physicians will need support from the industry to develop user-friendly platforms that streamline data visualization and analysis. Highly detailed neuromonitoring may lead to more aggressive interventions, which could, in turn, result in complications or prolonged ICU stays. Striking the right balance between intervention and observation will require careful clinical judgment. While neuromonitoring has shown promise in observational studies, rigorous randomized controlled trials will be necessary to validate the efficacy and safety of these technologies. Ethical considerations surrounding the use of experimental interventions in critically ill patients must also be addressed. Achieving true individualized care for every brain-injured patient may be challenging in real-world clinical settings. Identifying patients who require complex neuromonitoring and tailoring interventions accordingly will require careful patient selection and resource allocation. The collection and analysis of extensive patient data raise ethical and privacy concerns. Ensuring the secure storage and responsible use of patient information will be paramount.

## Conclusions

The future of neuromonitoring in severe adult brain-injured patients includes the use of several noninvasive or invasive techniques aiming at better understanding the consequences of the evolution of brain injury on cerebral compliance, oxygenation or metabolism. This approach will allow individualized care, implementing a personalized approach to patient care. Moreover, continuous neuromonitoring allows for the real-time assessment of dynamic changes in all these vital variables will help in identifying trends and guiding timely interventions. This could bring to early detection of secondary systemic and cerebral brain injuries and an adequate titration of therapies. Although of interest, this approach has some potential limitations and requires validation in future prospective trials. Also, realizing these promises will require a concerted effort in terms of knowledge acquisition, resource allocation, interdisciplinary collaboration and ethical considerations.

## Data Availability

Not applicable.
